# Supplementary material for: Incomplete removal of extracellular glutamate controls synaptic transmission and integration at a cerebellar synapse
Source: eLife. 2021 Feb 22;10:e63819. doi: 10.7554/eLife.63819 (PMC7935485; doi:10.7554/eLife.63819)
Supplement: Figure 1—figure supplement 2—source data 1. [file elife-63819-fig1-figsupp2-data1.docx]

| ***Parameter*** | ***Description*** | ***Value*** | ***Units*** |
| --- | --- | --- | --- |
| *K*b1 | Glutamate binding rate, 1st site | 800 | mM^-1^ ms^-1^ |
| *K*b2 | Glutamate binding rate, 2nd site | 600 | mM^-1^ ms^-1^ |
| *K*b3 | Glutamate binding rate, 3rd site | 400 | mM^-1^ ms^-1^ |
| *K*b4 | Glutamate binding rate, 4th site | 200 | mM^-1^ ms^-1^ |
|  |  |  |  |
| *K*u1 | Glutamate unbinding rate, 1st site | 30 | ms^-1^ |
| *K*u2 | Glutamate unbinding rate, 2nd site | 40 | ms^-1^ |
| *K*u3 | Glutamate unbinding rate, 3rd site | 60 | ms^-1^ |
| *K*u4 | Glutamate unbinding rate, 4th site | 80 | ms^-1^ |
|  |  |  |  |
| *K*d1 | Channel desensitization rate with 1 site bound | 0.25 | ms^-1^ |
| *K*d2 | Channel desensitization rate with 2 sites bound | 0.25 | ms^-1^ |
| *K*d3 | Channel desensitization rate with 3 sites bound | 1 | ms^-1^ |
| *K*d4 | Channel desensitization rate with 4 sites bound | 1 | ms^-1^ |
|  |  |  |  |
| *K*r1 | Channel resensitization rate with 1 site bound | 0.05 | ms^-1^ |
| *K*r2 | Channel resensitization rate with 2 sites bound | 0.05 | ms^-1^ |
| *K*r3 | Channel resensitization rate with 3 sites bound | 0.022 | ms^-1^ |
| *K*r4 | Channel resensitization rate with 4 sites bound | 0.022 | ms^-1^ |
|  |  |  |  |
| *K*o1 | Channel opening rate with 1 site bound | 3 | ms^-1^ |
| *K*o2 | Channel opening rate with 2 sites bound | 4 | ms^-1^ |
| *K*o3 | Channel opening rate with 3 sites bound | 4 | ms^-1^ |
| *K*o4 | Channel opening rate with 4 sites bound | 4 | ms^-1^ |
|  |  |  |  |
| *K*c1 | Channel closing rate with 1 site bound | 1.5 | ms^-1^ |
| *K*c2 | Channel closing rate with 2 sites bound | 1 | ms^-1^ |
| *K*c3 | Channel closing rate with 3 sites bound | 1 | ms^-1^ |
| *K*c4 | Channel closing rate with 4 sites bound | 1.5 | ms^-1^ |
|  |  |  |  |
|  |  |  |  |
| *O1* | Conductance with 1 site bound | 5 | pS |
| *O2* | Conductance with 2 site bound | 10 | pS |
| *O3* | Conductance with 3 site bound | 15 | pS |
| *O4* | Conductance with 4 site bound | 20 | pS |

Note that these rates are before the Q10 adjustment of 2.4. No temperature adjustment was made to conductances.
