# Supplementary material for: Incomplete removal of extracellular glutamate controls synaptic transmission and integration at a cerebellar synapse
Source: eLife. 2021 Feb 22;10:e63819. doi: 10.7554/eLife.63819 (PMC7935485; doi:10.7554/eLife.63819)
Supplement: Transparent reporting form [file elife-63819-transrepform.docx]

***eLife’s* transparent reporting form**

We encourage authors to provide detailed information *within their submission* to facilitate the interpretation and replication of experiments. Authors can upload supporting documentation to indicate the use of appropriate reporting guidelines for health-related research (see [EQUATOR Network](http://www.equator-network.org/%20)), life science research (see the [BioSharing Information Resource](https://biosharing.org/)), or the [ARRIVE guidelines](http://www.plosbiology.org/article/info:doi/10.1371/journal.pbio.1000412) for reporting work involving animal research. Where applicable, authors should refer to any relevant reporting standards documents in this form.

If you have any questions, please consult our Journal Policies and/or contact us: [editorial@elifesciences.org](mailto:editorial@elifesciences.org).

**Sample-size estimation**

- You should state whether an appropriate sample size was computed when the study was being designed
- You should state the statistical method of sample size computation and any required assumptions
- If no explicit power analysis was used, you should describe how you decided what sample (replicate) size (number) to use

Please outline where this information can be found within the submission (e.g., sections or figure legends), or explain why this information doesn’t apply to your submission:

Statistical methods were not used to predetermine sample size. Sample size was determined based on the variability across experiments. For whole-cell recordings, each cell was treated as an independent sample. Our sample sizes are similar to those reported in the field.

**Replicates**

- You should report how often each experiment was performed
- You should include a definition of biological versus technical replication
- The data obtained should be provided and sufficient information should be provided to indicate the number of independent biological and/or technical replicates
- If you encountered any outliers, you should describe how these were handled
- Criteria for exclusion/inclusion of data should be clearly stated
- High-throughput sequence data should be uploaded before submission, with a private link for reviewers provided (these are available from both GEO and ArrayExpress)

Please outline where this information can be found within the submission (e.g., sections or figure legends), or explain why this information doesn’t apply to your submission:

Detailed information can be found in the methods section. No outliers were left out.

**Statistical reporting**

- Statistical analysis methods should be described and justified
- Raw data should be presented in figures whenever informative to do so (typically when N per group is less than 10)
- For each experiment, you should identify the statistical tests used, exact values of N, definitions of center, methods of multiple test correction, and dispersion and precision measures (e.g., mean, median, SD, SEM, confidence intervals; and, for the major substantive results, a measure of effect size (e.g., Pearson's r, Cohen's d)
- Report exact p-values wherever possible alongside the summary statistics and 95% confidence intervals. These should be reported for all key questions and not only when the p-value is less than 0.05.

Please outline where this information can be found within the submission (e.g., sections or figure legends), or explain why this information doesn’t apply to your submission:

Statistical analyses are described within the results section and figure legends. t-tests were used to compare two groups. Paired t-tests were used in experiments that measured a dependent variable before and after some perturbation. ANOVAs, followed by Tukey’s multiple comparison tests were used when comparing across more than two groups and the data were adequately normally distributed and had equal variance. When the data did not meet the criteria for parametric tests, non-parametric tests were used. When comparing more than 2 groups, Friedman tests were used, followed by Dunn’s multiple comparison tests. When two groups were compared that were not normally distributed, Mann-Whitney tests were used. Scatter plots are used in all cases to show each data point in addition to the mean and sem or sd. Exact P values are reported.

(For large datasets, or papers with a very large number of statistical tests, you may upload a single table file with tests, Ns, etc., with reference to sections in the manuscript.)

**Group allocation**

- Indicate how samples were allocated into experimental groups (in the case of clinical studies, please specify allocation to treatment method); if randomization was used, please also state if restricted randomization was applied
- Indicate if masking was used during group allocation, data collection and/or data analysis

Please outline where this information can be found within the submission (e.g., sections or figure legends), or explain why this information doesn’t apply to your submission:

Experimental groups are defined in the results and methods sections. The experimenter was not blinded to the conditions (e.g. drugs applied).

**Additional data files (“source data”)**

- We encourage you to upload relevant additional data files, such as numerical data that are represented as a graph in a figure, or as a summary table
- Where provided, these should be in the most useful format, and they can be uploaded as “Source data” files linked to a main figure or table
- Include model definition files including the full list of parameters used
- Include code used for data analysis (e.g., R, MatLab)
- Avoid stating that data files are “available upon request”

Please indicate the figures or tables for which source data files have been provided:

All data points are represented in scatter plots. A python script will be included that was written to produce the sinusoidal stimulation pattern used in Figure 7. The AMPAR detailed kinetic model and the diffusion equation fitting tool will be made available in the NEURON database: https://senselab.med.yale.edu/ModelDB/
